# Salvianolic Acid B Protects Intervertebral Discs from Oxidative Stress-Induced Degeneration via Activation of the JAK2/STAT3 Signaling Pathway

**DOI:** 10.1155/2021/6672978

**Published:** 2021-02-12

**Authors:** Shouqian Dai, Ting Liang, Xiu Shi, Zongping Luo, Huilin Yang

**Affiliations:** ^1^Department of Orthopedics, The First Affiliated Hospital of Soochow University, Orthopedics Institute of Soochow University, Suzhou, Jiangsu, China; ^2^Department of Obstetrics and Gynecology, The First Affiliated Hospital, Soochow University, Suzhou, Jiangsu, China

## Abstract

**Objective:**

To evaluate the influence of salvianolic acid B (SAB), an antioxidant derived from Danshen, on intervertebral disc degeneration (IDD) and its possible molecular mechanisms.

**Methods:**

Sixty adult rats were randomly grouped (control, IDD, and SAB IDD groups). IDD was induced using needle puncture. The rats received daily administration of SAB (20 mg/kg) in the SAB IDD group while the other two groups received only distilled water. The extent of IDD was evaluated using MRI after 3 and 6 weeks and histology after 6 weeks. Oxidative stress was assessed using the ELISA method. In *in vitro* experiments, nucleus pulposus cells (NPCs) were treated with H_2_O_2_ (100 *μ*M) or SAB+H_2_O_2_, and levels of oxidative stress were measured. Cell apoptosis was assessed by flow cytometry, expression levels of Bcl-2, Bax, and cleaved caspase-3 proteins. Cell proliferation rate was assessed by EdU analysis. Pathway involvement was determined by Western blotting while the influence of the pathway on NPCs was explored using the pathway inhibitor AG490.

**Results:**

The data demonstrate that SAB attenuated injury-induced IDD and oxidative stress, caused by activation of the JAK2/STAT3 signaling pathway *in vivo*. Oxidative stress induced by H_2_O_2_ was reversed by SAB *in vitro*. SAB reduced the increased cell apoptosis, cleaved caspase-3 expression, and caspase-3 activity induced by H_2_O_2_. Reduced cell proliferation and decreased Bcl-2/Bax ratio induced by H_2_O_2_ were rescued by SAB. Additionally, the JAK2/STAT3 pathway was activated by SAB, while AG490 counteracted this effect.

**Conclusion:**

The results suggest that SAB protects intervertebral discs from oxidative stress-induced degeneration by enhancing proliferation and attenuating apoptosis via activation of the JAK2/STAT3 signaling pathway.

## 1. Introduction

Over recent years, lower back pain has been recognized as a common disorder that primarily afflicts the elderly, contributing considerably to socioeconomic burden [1–3]. The intervertebral disc (IVD) is important for stabilization and biomechanical maintenance of the spinal column. IVD degeneration (IDD) is believed to be a principal cause of lower back pain, and more than eighty percent of adults have at some point suffered IDD [4]. Therefore, the search for treatments that would be effective for IDD has gained considerable attention. Even now, the precise pathogenesis of IDD remains unknown, although the possible etiology includes genetic factors, trauma, infection, aging, smoking, and mechanical stress [5]. The characteristic changes in IDD are decreased intervertebral space, apoptosis of nucleus pulposus cells (NPCs), loss of nucleus pulposus (NP) collagen, and vertebral endplate calcification [6]. The central part of an adult IVD, the nucleus pulposus (NP), is primarily composed of chondrocyte-like NP cells (NPCs) that produce extracellular matrix molecules that stabilize the biomechanical equilibrium and structure of the IVD [7]. As IDD occurs and progresses, the NPCs commonly become significantly functionally impaired due to either increased apoptosis or decreased proliferation [8]. Previous studies have demonstrated that oxidative stress may perform a crucial role in the pathology of IDD [9–11].

It has been reported that significant structural alterations in degenerated IVDs at least partly result from oxidative stress resulting from excessive accumulation of reactive oxygen species (ROS) [12]. In physiological conditions, the production and elimination of cellular ROS are in dynamic equilibrium. Oxidative stress occurs where this equilibrium is out of balance, for example, in the microenvironment of degenerated IVDs [13, 14]. A number of oxidative stress markers have been identified in human degenerated discs, including peroxynitrite, glutathione (GSH), superoxide dismutase 2 (SOD2), malondialdehyde (MDA), and advanced glycation end products (AGEs) [15–17]. Additionally, SOD2 activity in the serum of patients and rats with IDD has been found to be significantly reduced, while a number of markers such as MDA, peroxide, and NO are significantly increased [18]. The observations above demonstrate that ROS and oxidative stress may significantly influence the pathological development of IDD.

Previous studies have shown that the herbal drug Danshen has a number of pharmacological and clinical effects [19]. The constituents of Danshen are principally water-soluble phenolic acids and fat-soluble tanshinone compounds [20–22]. Salvianolic acid B (SAB) is the most abundant water-soluble compound extracted from Danshen. Previous studies have confirmed that SAB has antioxidative properties and eliminates superoxide anion radicals (·O_2_^−^), thereby inhibiting hydrogen peroxide- (H_2_O_2_-) induced apoptosis [23, 24]. It has also been reported that SAB can increase the proliferation of human umbilical vein endothelial cells [25]. Chang et al. reported that SAB was effective in scavenging excess production of ROS, and this antioxidant activity was stronger than even glutathione or vitamin E [26]. Hence, it can be deduced that SAB may help attenuate oxidative stress-induced IDD. However, few studies have been conducted in this field, and thus the precise influence of SAB on degenerated IVDs or NPCs, and its underlying mechanisms remain unresolved.

The Janus kinase 2 (JAK2)/signal transducer and activator of transcription 3 (STAT3) pathway is a common pathway of signal transduction for multiple cytokines [27–29] such as TNF-*α*, IL-6, and IL-1*β*, which transmits signals from the cell membrane directly to the nucleus to initiate gene transcription [30], occurring in inflammation and oxidative stress. Additionally, the JAK2/STAT3 signaling pathway has been shown to regulate the catabolism of genes related to degenerated disc NPCs [31]. From this, we have reason to infer that the degeneration of IVDs and NPCs is closely associated with the JAK2/STAT3 signaling pathway. However, whether the JAK2/STAT3 pathway is involved in the influence of SAB on IDD remains unclear.

Therefore, the present study is aimed at evaluating the protective effects of SAB on IVD in SD rats with injury-induced IDD *in vivo* and by use of an *in vitro* model of H_2_O_2_-induced IDD in NPCs. Furthermore, the signaling pathway involved in the attenuation of IDD by SAB was investigated. This research may facilitate advances in the clinical use of SAB for treatment of degenerative disease.

## 2. Methods and Materials

### 2.1. Reagents

SAB was obtained from ChromaDex, Inc. (Irvine, CA, USA), dissolved in distilled water, and stored at -20°C prior to use. Assays to quantify GSH, SOD2, MDA, and ROS and caspase-3 activity kits were acquired from Beyotime Biotechnology Co. Ltd. (Shanghai, China). Dulbecco's modified Eagle medium (DMEM)/F12 medium and fetal bovine serum (FBS) were purchased from Invitrogen Inc. (MD, USA). All primary antibodies used in the study were obtained from Cell Signaling Technology, Inc. (MA, USA). The secondary antibodies were from Beyotime Biotechnology Co. Ltd. (Shanghai, China). Sigma-Aldrich Inc. (St. Louis, MO, USA) supplied all other reagents.

### 2.2. In Vivo Study

#### 2.2.1. Animal Protocol

Sixty adult male Sprague-Dawley rats were purchased from the animal center at Soochow University (Suzhou, China). Animals were maintained under normal conditions and randomly allocated into three equal groups (control, IDD, and SAB IDD) prior to surgery. All rats received treatments, as appropriate, for two weeks and then were subjected to experimental surgery, as described below. All animal experiments were approved by the Animal Care and Experiment Committee of Soochow University (2019 Approval No. ECSU-2019000210).

#### 2.2.2. IDD Model Induction

Percutaneous needle puncture has been shown to be an effective method of induction of disc degeneration [32]. After acclimatization, animals were anesthetized by inhalation of 2% fluothane in oxygen/nitrous oxide. The surgical procedure was performed on the vertebrae in the tail, as described previously [33]. In the IDD and SAB IDD groups, the Co8-9 discs were punctured using a 20-gauge needle. Full penetration and sham surgery were performed on rats in the control group. Following surgery, all rats in the SAB IDD group received SAB (20 mg/kg) by oral gavage once per day for six consecutive weeks, consistent with the dose used in a previous study [34]. Only distilled water was administrated to rats in the other two groups. After 6 weeks, all rat tails were scanned by MRI under isoflurane anesthesia and then the Co8-9 discs were collected for subsequent experiments. All experimental steps complied with the Animal Research Reporting of In Vivo Experiments (ARRIVE) guidelines.

#### 2.2.3. Magnetic Resonance Imaging (MRI) Examination

T2 mapping of MRI signal intensity is commonly used to measure the degree of IDD. The procedure was conducted as previously described, in a 1.5 T MRI scanner (GE, USA) [35]. Briefly, all rat IVDs were scanned at the 3- and 6-week time points. The T2 signal intensity of Co8-9 discs was recorded, and the ratio of T2 signal intensity of the injured to control discs was calculated then analyzed using the ImageJ software. Therefore, normalized disc intensity was presented as values between 0 and 1.

#### 2.2.4. Histological Evaluation

All target IVDs collected were fixed in 10% formalin and embedded in paraffin. Then, 5 *μ*m serial sections were prepared from the midsagittal region. Hematoxylin and eosin (H&E), Safranin-O Fast green, and alcian blue staining were used to identify histological changes in the IVDs and assessed using a previously described grading scale [33], providing scores from 5 to 15 points, representing IVDs that were normal to severely degenerated [33].

#### 2.2.5. Biochemical Analysis

The collected Co8-9 discs were digested by papain at 65°C for 1 hour then centrifuged for 10 min. GSH concentration was then measured using a glutathione assay kit, in accordance with standard experimental procedures. A lipid peroxidation MDA assay kit was used to calculate MDA concentration. SOD2 levels were measured using an ELISA, in accordance with standard protocols.

#### 2.2.6. Western Blotting

Expression levels of JAK2, p-JAK2, STAT3, and p-STAT3 in the discs were tested using routine Western blotting analysis. After blocking in 5% nonfat milk, the membranes were incubated with the following anti-rat primary antibodies at 4°C overnight: JAK2 (1 : 1000), p-JAK2 (1 : 1000), STAT3 (1 : 1000), p-STAT3 (1 : 1000), and GAPDH (1 : 2000), after which they were incubated with an HRP-conjugated secondary antibody at 37°C for one hour. Finally, the relative optical densities of all bands were normalized to GAPDH.

### 2.3. In Vitro Study

#### 2.3.1. NPC Isolation and Culture

Normal IVDs were obtained from rat tails and cut into small pieces. Firstly, the coccygeal spinal columns (Co6-Co10) of rats were separated and the NP of each disc collected under aseptic conditions. NPCs were obtained using a dissecting microscope in accordance with methods reported previously [36]. The NPCs were then cultured in DMEM/F12 medium supplemented with 10% FBS and 1% penicillin/streptomycin in a conventional culture environment. After cell cultures were confluent, the NPCs were subcultured and cells from the second passage were used in the *in vitro* studies. All protocols were conducted in accordance with the relevant guidelines of the Ethics Committee.

#### 2.3.2. Cell Treatment

As previously described, H_2_O_2_ (100 *μ*M) was used to induce oxidative damage [37]. Untreated NPCs were used as controls. An MTT assay was performed using different concentrations of SAB (0.001, 0.01, 0.1, 1, 10, and 100 nM) to determine the concentration most appropriate for use in the following experiments, as previously described [38]. To evaluate the effects of SAB on apoptosis, proliferation, and oxidative stress, NPCs were randomized into three groups: control, H_2_O_2_, and SAB+H_2_O_2_ groups (1 nM SAB for 24 h, then 100 *μ*M H_2_O_2_ for 24 h). To determine the signaling pathway targeted by SAB, the NPCs were divided into four groups: control, H_2_O_2_, SAB+H_2_O_2_, and AG490+SAB+H_2_O_2_ (1 nM SAB for 24 h, 40 *μ*M AG490 for 24 h, and then 100 *μ*M H_2_O_2_ for 24 h).

#### 2.3.3. Identification of NPCs

Immunocytochemistry and immunofluorescence were used to identify the NPCs obtained from IVD tissue. NPCs were fixed in 4% paraformaldehyde for 20 min at 37°C, permeabilized in 0.2% Triton X-100, and blocked using goat serum. The NPCs were incubated with primary antibodies against collagen II and cytokeratin 19 at 4°C overnight. The NPCs were then incubated with an HRP-conjugated (for immunocytochemistry) or fluorescein isothiocyanate- (FITC-) labeled (for immunofluorescence) secondary antibody. Images were acquired using light or fluorescence microscopy.

#### 2.3.4. Intracellular ROS Measurement

Briefly, after the treatment appropriate for their grouping, NPCs were incubated with DCFH-DA for half an hour then washed three times. ROS concentration of the NPCs was determined using a ROS assay kit in accordance with the manufacturer's instructions. Finally, ROS concentration, presented as relative fluorescence units (RFU), was calculated from fluorescence intensity at wavelengths of 490/585 nm.

#### 2.3.5. Measurement of GSH, SOD2, and MDA

Intracellular levels of GSH, SOD2, and MDA, which reflect oxidation state, were measured using the corresponding assay kit (Beyotime Biotechnology Co. Ltd., Shanghai, China) in accordance with the manufacturer's instructions. The NPCs were first digested with 0.25% trypsin and centrifuged for 15 minutes at 4°C at 900 g. The sample supernatants and standards were prepared in cuvettes, and the OD values at 530 nm were recorded. GSH, SOD2, and MDA concentrations were obtained by reference to the standard curve. The data are presented as means ± SD of three replicate experiments.

#### 2.3.6. Flow Cytometry

The rate of apoptosis of NPCs was determined using a classic Annexin V-FITC/PI staining kit. Briefly, NPCs were collected and washed with PBS three times. NPCs were then resuspended and incubated with Annexin V-FITC and propidium iodide (PI) at 37°C in a dark room for 20 minutes. Finally, cell apoptosis was determined using a flow cytometer (BD Co., USA). Cells staining positive for Annexin V and negative for PI were considered apoptotic NPCs.

#### 2.3.7. Western Blotting

The expression levels of Bcl-2, Bax, cleaved caspase-3, JAK2, p-JAK2, STAT3, and p-STAT3 in NPCs were measured using Western blotting, as described in the protocol above (Section 2.2.6). All primary antibodies were diluted 1 : 1000. Relative expression levels were normalized to GAPDH.

#### 2.3.8. Measurement of Caspase-3 Activity

Caspase-3 activity was evaluated using an activity assay kit in accordance with standard protocols. Briefly, NPCs in culture medium were collected, lysed in RIPA lysis buffer, and then mixed with the appropriate reaction reagents at room temperature. Optical density (OD) was measured at a wavelength of 405 nm, and caspase-3 activity was calculated by reference to a standard curve then normalized to total protein concentration.

#### 2.3.9. NPC Proliferation Assay

Cell proliferation in different groups was measured by staining with the thymidine analog 5-ethynyl-2deoxyuridine (EdU). Briefly, NPCs (1 × 10^6^) were seeded in each well of 6-well plates then treated with EdU (10 *μ*M) conjugated with Alexa-Fluor 594 (Alexa-594, Invitrogen) at 37°C in a dark room for 30 minutes. Cells were then counterstained with DAPI then mounted. Images were acquired using a Leica fluorescence microscope. The ratio of EdU-positive cells/total cells was obtained, and the rate of cell proliferation in the different groups compared.

### 2.4. Statistical Analyses

All data are presented as means ± SD. One-way analysis of variance (ANOVA) was used to compare multiple groups after verification of normality with post hoc comparisons using a least-squares difference (LSD) method. Statistical analyses were performed using the SPSS v20 statistical software (SPSS Inc., Chicago, IL, USA). *P* values <0.05 were considered statistically significant.

## 3. Results

### 3.1. SAB Attenuates Injury-Induced IDD and Oxidative Stress and Activates the JAK2/STAT3 Signaling Pathway In Vivo

**Figure 1:**
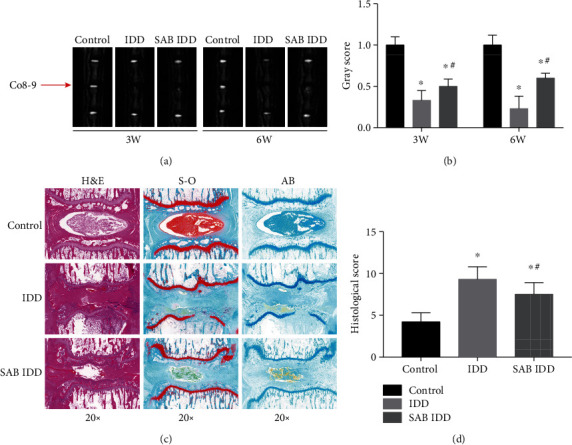
SAB reversed the reduced T2-weighed MR signal intensity induced by injury to IVDs and rescued the consequent disc degeneration *in vivo*. (a) MRI was used to detect changes in MR signal intensity of the Co8-9 discs in each group after 3 and 6 weeks. (b) Quantitative analysis demonstrated that a significant decrease of T2 signal intensity was found in injured IVDs. This decrease could be both reversed by treatment with SAB. (c) H&E staining displayed representative histopathological changes to IVDs in each group. IVDs in the control group exhibited a normal structure. In the IDD group, the border between the AF and NP was clearly disrupted, the NPCs becoming separated by extracellular proteoglycan matrix and the NP having almost disappeared. In the SAB IDD group, the characteristics of degeneration were largely reversed by treatment with SAB. (d) Induction of IDD resulted in significantly higher histological scores compared with the control group. SAB treatment significantly reduced the increased histological score. ^∗^*P* < 0.05 compared with the control group; ^#^*P* < 0.05 compared with the IDD group. SAB: salvianolic acid B; IDD: intervertebral disc degeneration; IVD: intervertebral disc; NP: nucleus pulposus; AF: annulus fibrosus; H&E: hematoxylin and eosin; S-O: Safranin-O Fast green; AB: alcian blue.

T2-weighted signal intensities of the MRI examination of Co8-9 IVDs were markedly lower in the IDD group than that of the control group after both 3 and 6 weeks. However, treatment with SAB in the SAB IDD group clearly reversed this decrease (Figures 1(a) and 1(b)). Histological staining indicated that the IVD in the control group had a normal structure but the border between the annulus fibrosus (AF) and nucleus pulposus (NP) in the IDD group was clearly disrupted. NPCs had become separated by an extracellular proteoglycan matrix, and the NP had almost disappeared. The AF became disorganized due to inward bulging of the inner annulus. However, SAB significantly reduced the injury induced by IDD and partially reestablished the structure of the IVD (Figures 1(c) and 1(d)).

**Figure 2:**
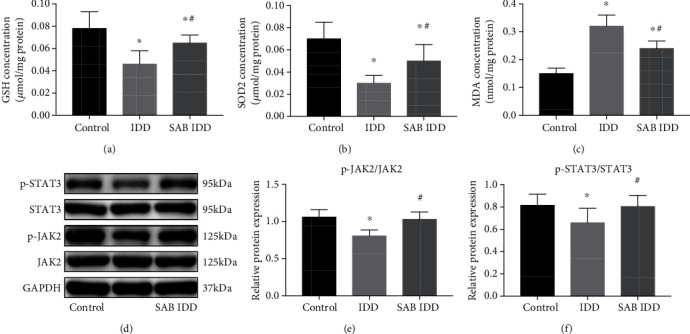
SAB reversed the effects on the antioxidant system induced by puncture injury *in vivo* that activated the JAK2/STAT3 signaling pathway. Animals were divided into three groups: control group, IDD group, or SAB group. Concentrations of (a) GSH, (b) SOD2, (c) and MDA were measured by assay kits. (d) Expression levels of phosphorylated and total JAK2 and STAT3 in IVDs were measured by Western blotting, and the relative ratios of (e) p-JAK2/JAK2 and (d) p-STAT3/STAT3 were calculated from gray-level values. ^∗^*P* < 0.05 compared with the control group; ^#^*P* < 0.05 compared with the IDD group. SAB: salvianolic acid B; GSH: glutathione; SOD2: superoxide dismutase 2; MDA: malondialdehyde; GAPDH: glyceraldehyde-3-phosphate dehydrogenase; JAK2: Janus kinase 2; STAT3: signal transducer and activator of transcription 3.

Figures 2(a) and 2(b) demonstrate that GSH and SOD2 levels in the IVD of the IDD group decreased markedly compared with the control group, and SAB medication significantly reversed this change.  Figure 2(c) shows that SAB treatment significantly decreased the levels of MDA that had been induced by disc injury in the IDD group. Western blotting demonstrated that the phosphorylation levels of JAK2 and STAT3 in the IDD group were significantly lower than in the control group. However, SAB clearly upregulated the expression levels of p-JAK2 and p-STAT3 in rats in the IDD group (Figures 2(d)–2(f)). These results suggest that SAB inhibits disc degeneration and oxidative stress *in vivo*, probably via the JAK2/STAT3 signaling pathway.

### 3.2. Viability Assays for SAB-Treated NPCs and Cell Identification

**Figure 3:**
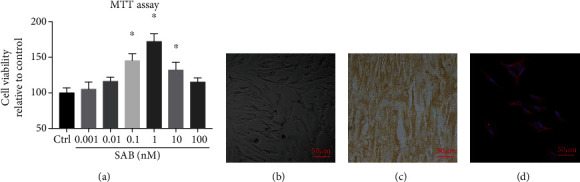
Cell viability in NPCs treated with SAB and identification of NPCs. (a) MTT assay of NPCs treated with different concentrations of SAB. (b) Morphology of NPCs by phase-contrast microscopy. (c) Immunocytochemistry showing the expression of collagen II in NPCs. (d) Immunofluorescence demonstrating the expression of cytokeratin 19 in NPCs. Scale bars = 50 *μ*m. ^∗^*P* < 0.05 compared with the control group. SAB: salvianolic acid B; NPCs: nucleus pulposus cells.

MTT assays were used to investigate the viability of NPCs incubated with different concentrations of SAB (0.001, 0.01, 0.1, 1, 10, and 100 nM). The data indicate that cell viability in the 0.1, 1, and 10 nM groups was clearly greater than other concentrations, 1 nM being the most significant (Figure 3(a)). Thus, 1 nM SAB was selected for subsequent experiments.

Normal NPCs exhibited an appearance similar to chondrocytes under phase-contrast microscopy (Figure 3(b)). Immunocytochemical staining indicated that collagen II was expressed within the cytoplasm (Figure 3(c)) while immunofluorescence demonstrated the expression of the molecular marker cytokeratin 19 in the NPCs (Figure 3(d)).

### 3.3. SAB Inhibits H_2_O_2_-Induced Oxidative Stress and Apoptosis In Vitro

**Figure 4:**
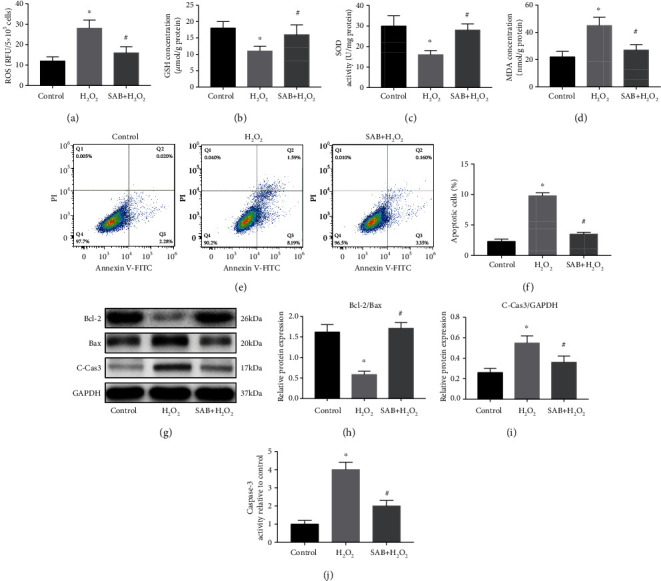
SAB attenuated H_2_O_2_-induced oxidative stress and apoptosis of NPCs *in vitro*. (a) H_2_O_2_-induced production of ROS was decreased by treatment with SAB. (b–d) Increased oxidative stress (decreased GSH and SOD2, and increased MDA) in the H_2_O_2_ group was reversed by SAB. (e, f) Flow cytometry assay demonstrated that SAB decreased H_2_O_2_-induced NPC apoptosis. (g–i) Western blotting showed an elevated ratio of Bcl-2/Bax and decreased level of cleaved caspase-3 in the SAB group compared with the H_2_O_2_ group. (j) Increased caspase-3 activity in the H_2_O_2_ group was inhibited by treatment with SAB. ^∗^*P* < 0.05 compared with the control group; ^#^*P* < 0.05 compared with the H_2_O_2_ group. SAB: salvianolic acid B; ROS: reactive oxygen species; GSH: glutathione; SOD2: superoxide dismutase; MDA: malondialdehyde; NPCs: nucleus pulposus cells; GAPDH: glyceraldehyde-3-phosphate dehydrogenase; C-Cas3: cleaved caspase-3.

ROS, GSH, SOD2, and MDA levels were assessed to reflect the degree of oxidative stress. Figures 4(a)–4(d) demonstrate that in the H_2_O_2_ group, ROS and MDA levels were clearly less, and GSH and SOD2 levels significantly higher than those of the control group. In the SAB group, however, ROS, GSH, SOD2, and MDA levels were restored, indicating that SAB was able to inhibit the antioxidant system *in vitro*.

Apoptosis of NPCs was evaluated by flow cytometry, Western blotting, and ELISA. The flow cytometry results indicate that NPC apoptosis in the H_2_O_2_ group was significantly higher than in the control group, an increase that was reversed by SAB (Figures 4(e) and 4(f)). Similar changes were also observed by Western blot analysis, where the ratio of Bcl-2/Bax increased and cleaved caspase-3 decreased in the SAB H_2_O_2_ group (Figures 4(g)–4(j)). Therefore, we propose that SAB inhibited H_2_O_2_-induced NPC apoptosis.

### 3.4. SAB Promotes NPC Proliferation and Activates the JAK2/STAT3 Signaling Pathway In Vitro

**Figure 5:**
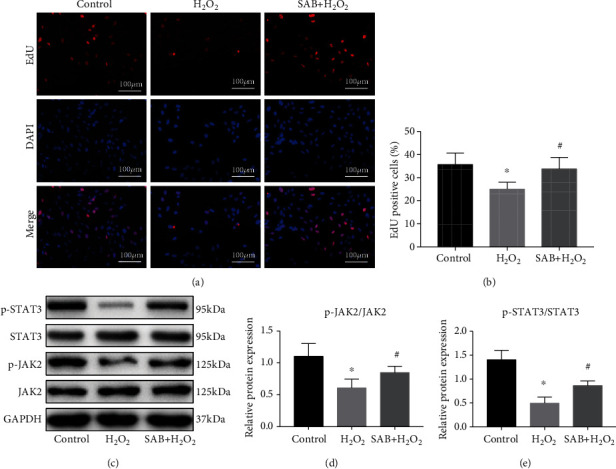
SAB promoted proliferation of NPCs reduced by H_2_O_2_*in vitro* via activation of the JAK2/STAT3 signaling pathway. (a, b) EdU assay showed that H_2_O_2_-induced downregulation of NPC proliferation was reversed by treatment with SAB. (c) JAK2 and p-JAK2 and STAT3 and p-STAT3 were investigated by Western blotting. Expression levels of (d) p-JAK2/JAK2 and (e) p-STAT3/STAT3 increased by SAB in H_2_O_2_-injured NPCs. Scale bars = 100 *μ*m. ^∗^*P* < 0.05 compared with the control group; ^#^*P* < 0.05 compared with the H_2_O_2_ group. SAB: salvianolic acid B; NPCs: nucleus pulposus cells; GAPDH: glyceraldehyde-3-phosphate dehydrogenase; JAK2: Janus kinase 2; STAT3: signal transducer and activator of transcription 3.

The EdU assay demonstrated that SAB clearly increased the proliferation of NPCs that had been reduced by H_2_O_2_ (Figures 5(a) and 5(b)). Western blotting demonstrated that treatment with SAB significantly increased the expression levels of p-JAK2 and p-STAT3, which were markedly lower in the H_2_O_2_ group (Figures 5(c)–5(e)). These data indicate that SAB was able to inhibit H_2_O_2_-induced oxidative stress and apoptosis, promote cell proliferation, and activate the JAK2/STAT3 pathway *in vitro*.

### 3.5. SAB Inhibits H_2_O_2_-Induced Disc Degeneration via the JAK2/STAT3 Signaling Pathway

**Figure 6:**
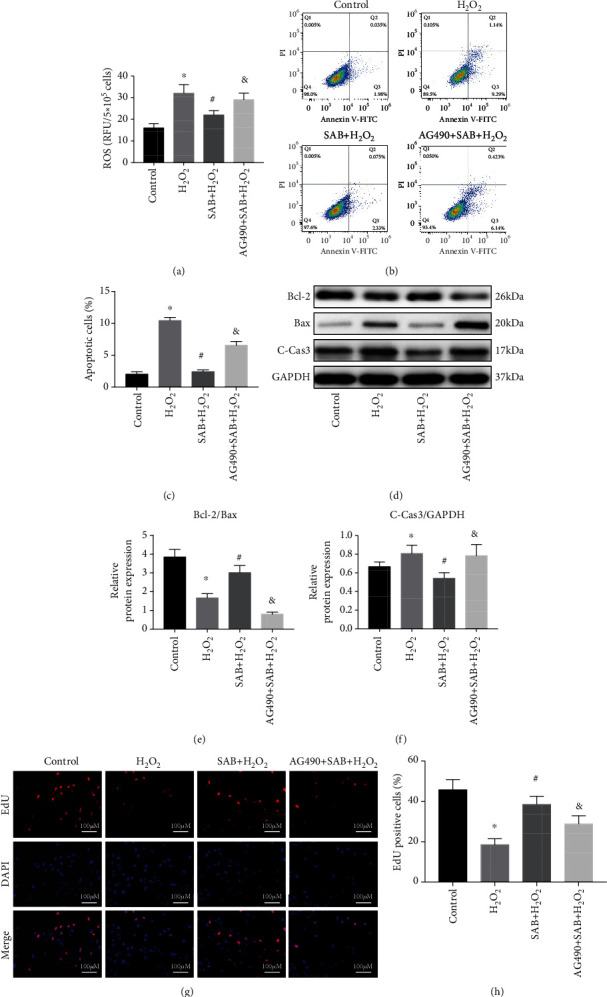
SAB reduced H_2_O_2_-induced ROS production, ameliorated H_2_O_2_-induced NPC apoptosis, and promoted H_2_O_2_-reduced proliferation of NPCs via the JAK2/STAT3 pathway. (a) Inhibitory effects of SAB on H_2_O_2_-induced ROS production were abrogated by AG490. (b, c) Flow cytometry demonstrated that the inhibitory effects of SAB on H_2_O_2_-induced NPCs apoptosis were abrogated by AG490. (d) Apoptosis-associated proteins assessed by Western blotting. Effects of SAB on H_2_O_2_-induced (e) Bcl-2/Bax and (f) cleaved caspase-3 were abrogated by AG490. (g, h) EdU assay demonstrated that the effects of SAB on NPC proliferation were downregulated by AG490 treatment. Scale bars = 100 *μ*m. ^∗^*P* < 0.05 compared with the control group; ^#^*P* < 0.05 compared with the H_2_O_2_ group; ^&^*P* < 0.05 compared with the SAB+H_2_O_2_ group. SAB: salvianolic acid B; NPCs: nucleus pulposus cells; ROS: reactive oxygen species; C-Cas3: cleaved caspase-3; GAPDH: glyceraldehyde-3-phosphate dehydrogenase.

**Figure 7:**
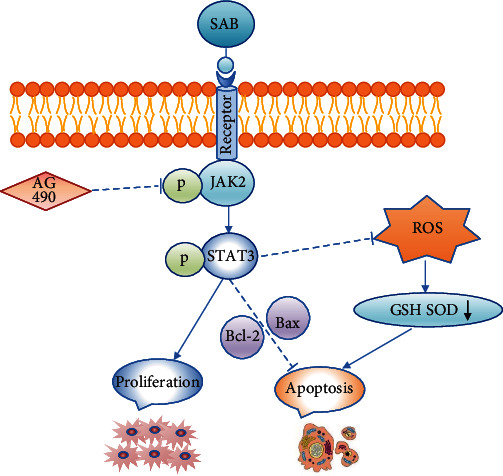
Model of involvement of SAB in enhancing proliferation and attenuating oxidative stress and apoptosis through activation of the JAK2/STAT3 signaling pathway. SAB: salvianolic acid B; ROS: reactive oxygen species; JAK2: Janus kinase 2; STAT3: signal transducer and activator of transcription 3; GSH: glutathione; SOD2: superoxide dismutase.

To investigate whether SAB influences oxidative stress, apoptosis, and proliferation of NPCs through the JAK2/STAT3 signaling pathway, the JAK2 antagonist AG490 was utilized. The data indicate that SAB inhibited H_2_O_2_-induced oxidative stress and apoptosis in NPCs. However, this inhibitory behavior was markedly neutralized by the addition of AG490 (Figures 6(a)–6(c)). In addition, treatment with SAB also rescued changes in expression levels of Bcl-2, Bax, and cleaved caspase-3, and this alteration was abrogated by AG490 (Figures 6(d)–6(f)). Similarly, SAB increased H_2_O_2_-reduced cell proliferation, and this effect was also abrogated by AG490 (Figures 6(g) and 6(h)). Taken together, we conclude that SAB inhibits disc degeneration via the JAK2/STAT3 signaling pathway (Figure 7).

## 4. Discussion

In the present study, we investigated the effects of SAB on IDD. The results demonstrate that SAB reduced IVD degeneration *in vivo*, inhibited oxidative stress and apoptosis of NPCs, and promoted cell proliferation in an *in vitro* model of IDD. Further mechanistic investigation indicated that the JAK2/STAT3 signaling pathway was involved in the influence of SAB on IDD. The results demonstrate that SAB may represent a potential treatment for degenerative disc disease.

Disc degeneration is an important cause of lower back pain and disability [2]. Few effective therapies are available to ease or reverse the course of IDD. Therefore, the present study explored a strategy to prevent the progression of IDD. SAB, a natural polyphenolic ingredient rich in Danshen, has multiple pharmacological properties, such as being an antioxidant and anti-inflammatory agent [39, 40]. It has recently been reported that SAB combined with mesenchymal stem cells is able to repair degenerated IVDs more effectively than stem cells alone [41]. However, whether SAB can inhibit the progression of IDD remains controversial. Therefore, the present study first examined the influence of SAB on injury-induced IDD in rats. The data indicate that needle puncture resulted in decreased T2-weighted signal intensity from the IVDs and altered disc morphology. Treatment with SAB significantly attenuated injury-induced IDD and restored changes to IVD structure.

It has previously been demonstrated that SAB can effectively protect the heart from ischemia-reperfusion injury [42]. SAB both increased and decreased cell viability, depending on the concentration. It has been reported that SAB promotes cell growth and inhibits the dedifferentiation of articular chondrocytes [43]. According to the MTT assay results, a SAB concentration of 1 nM most effectively promoted NPC viability. Similar results were also observed in previous studies involving different cell types [42]. We, therefore, used SAB at a concentration of 1 nM for subsequent *in vitro* experiments. Our findings demonstrate that H_2_O_2_ significantly inhibited the proliferation of NPCs while treatment with SAB clearly rescued that reduction in cell proliferation. This indicates that SAB can attenuate disc degeneration, at least partly through the promotion of NPC proliferation.

Oxidative stress has been reported to play a critical role in a number of physiological and pathological processes, activating a variety of signaling pathways, resulting in cell apoptosis or hyperplasia [44]. Oxidative stress that induces excessive ROS production can injure DNA and cells, while ROS scavengers will inhibit the cell apoptosis resulting from a variety of causes [45, 46]. Many previous studies suggest that oxidative stress plays a crucial role in the pathology of IDD [9, 10, 47], and oxidative stress-associated markers are significantly increased in degenerated human IVDs [15–17]. Cell apoptosis and injury can be markedly inhibited by antioxidants via radical-scavenging mechanisms [48]. Further studies have shown that the antioxidant capacity and scavenging activity of SAB were both greater than vitamin C [49]. The quantity of ROS production in microglial cells induced by lipopolysaccharide (LPS) was shown to be inhibited by SAB in a dose-dependent manner [50], suggesting that SAB is able to prevent oxidative stress. Our data demonstrate that the antioxidant system was weakened by injury or H_2_O_2_, while the situation could be rescued by treatment with SAB both *in vivo* and *in vitro*. The results of the present study were also consistent with other previous reports [51, 52]. Thus, SAB provides a protective effect against oxidative stress by regulating the antioxidant system.

It was demonstrated many years ago that apoptosis participates in IDD, large numbers of IVD cells undergoing programmed cell death [53]. Excessive apoptosis of NPCs reflects evident cellular and biochemical changes which occur during IDD [54, 55]. It has been reported in previous studies that external stimuli result in oxidative damage and increased apoptosis of NPCs [56–58]. H_2_O_2_ is commonly used to induce cell injury in *in vitro* experiments [59]. Therefore, we induced oxidative damage using H_2_O_2_ and investigated whether SAB can influence the apoptosis induced in NPCs by oxidative stress. In the present study, oxidative damage induced by 100 *μ*M H_2_O_2_ resulted in significantly increased cell apoptosis. Notably, SAB treatment suppressed H_2_O_2_-induced NPC apoptosis, promoted Bcl-2 expression, and inhibited the expression of Bax, thus rescuing the Bcl-2/Bax protein ratio in the SAB+H_2_O_2_ group. Additionally, *in vitro* cleaved caspase-3 protein expression and caspase-3 activity were decreased substantially by SAB. This suggests that SAB has the potential to treat IDD by preventing NPC apoptosis in conditions of oxidative stress. A previous study reported that SAB protected against dopamine-induced apoptosis in SH-SY5Y cells [60]. This protective effect of SAB appears to be due to its antioxidative potential. Additionally, another study demonstrated the protective effect of SAB against ox-LDL-induced HUVEC injury and apoptosis [61]. Finally, it was demonstrated that SAB protected vascular endothelial cells against oxidative stress-induced injury [28]. These findings further support the present observations.

Based on the previous findings, the underlying molecular mechanism and pathways of the IDD-attenuating properties of SAB were further explored. In the present study, the expression levels of p-JAK2 and p-STAT3 proteins in the SAB group were higher than those in the corresponding model group. However, after the addition of AG490, the p-JAK2 and p-STAT3 protein levels were clearly reduced. These findings demonstrate that the JAK2/STAT3 pathway can be activated by SAB, and treatment with the JAK2 antagonist AG490 significantly abrogated inhibition of apoptosis and promotion of proliferation by SAB. According to Liu et al. [62], SAB is able to maintain stem cell pluripotency via the JAK2/STAT3 signaling pathway. Liu et al. demonstrated that articular cartilage degeneration in osteoarthritis can be rescued by treatment with Danshen (that contains SAB), both *in vivo* and *in vitro* via activation of the JAK2/STAT3 pathway [62]. Furthermore, SMND-309, a novel derivative of SAB, has been shown to protect rat brains from ischemia and reperfusion damage via the JAK2/STAT3 signaling pathway. However, it should also be noted that the JAK2/STAT3 signaling pathway is probably not the only signaling pathway by which SAB attenuates IDD. It has previously been shown that SAB maintained stem cell pluripotency and promoted cell proliferation via activation of the JAK2/STAT3 pathway, in addition to the epidermal growth factor receptor-(EGFR-) extracellular signal-regulated kinase 1/2 (ERK1/2) pathways [62]. However, further evidence is required. The JAK2-STAT3 signaling pathway consists of three main components, namely, tyrosine kinase associated receptor (TKAR), JAK2, and STAT3. The receptors of multiple cytokines such as interleukins, growth hormones, epidermal growth factor, platelet-derived growth factor, and interferon are present on cell membranes. The binding of such a ligand to its corresponding receptor can activate JAK2, STAT3, and other target proteins that initiate the transcription of target genes representing the transmission of a biological signal from the extracellular environment to the intracellular biochemical systems. As a water-soluble substance, we speculate that SAB may also initiate a signaling cascade to achieve its multiple biological functions through combining with the cell membrane receptor, TKAR. However, reports of such aspects have not so far been found. Therefore, we will focus on the SAB receptor and its target genes in subsequent experiments to fully elucidate the complete signaling pathway. Together with the aforementioned findings, the present study demonstrates that SAB attenuates NPC apoptosis induced by oxidative damage and promotes cell proliferation via regulation of the JAK2/STAT3 signaling pathway.

However, there remain some limitations to the study. Firstly, we recognize that the protective effects of SAB on IDD require additional confirmation, especially in high-level animals or even humans before it can be used clinically. Secondly, the optimal therapeutic dose requires additional study, if possible. Thirdly, to further illustrate the molecular mechanism by which SAB inhibits IDD, additional research is required.

In conclusion, the results of the research suggest that SAB can reduce IVD degeneration *in vivo*, inhibit oxidative stress and apoptosis of NPCs, and promote cell proliferation in an IDD model *in vitro*. Additional study indicated that activation of the JAK2/STAT3 pathway is involved in the pathology of IDD. Taken together, it can be concluded that SAB ameliorates IDD by activating the JAK2/STAT3 signaling pathway. The results suggest that SAB may represent a potential treatment for disc degeneration.

## Data Availability

The data used to support the findings of this study are available from the corresponding author upon request.
