# Corrigendum to “Salvianolic Acid B Protects Intervertebral Discs from Oxidative Stress-Induced Degeneration via Activation of the JAK2/STAT3 Signaling Pathway”

**DOI:** 10.1155/2022/9874240

**Published:** 2022-02-23

**Authors:** Shouqian Dai, Ting Liang, Xiu Shi, Zongping Luo, Huilin Yang

**Affiliations:** ^1^Department of Orthopedics, The First Affiliated Hospital of Soochow University, Orthopedics Institute of Soochow University, Suzhou, Jiangsu, China; ^2^Department of Obstetrics and Gynecology, The First Affiliated Hospital, Soochow University, Suzhou, Jiangsu, China

In the article titled “Salvianolic Acid B Protects Intervertebral Discs from Oxidative Stress-Induced Degeneration via Activation of the JAK2/STAT3 Signaling Pathway” [1], there was an error in Section 3.3, and the following statement should be corrected:

“Figures 4(a)–4(d) demonstrate that in the H2O2 group, ROS and MDA levels were clearly less, and GSH and SOD2 levels significantly higher than those of the control group” should be corrected to

“Figures 4(a)–4(d) demonstrate that in the H2O2 group, ROS and MDA levels were clearly higher and GSH and SOD2 levels significantly lower than those of the control group.”

Additionally in Figure 2(d), the label for IDD was accidentally omitted during the typesetting process, and the corrected figure is as follows:

The authors confirm that this does not affect the results and conclusions of the article, and the editorial board agrees to the publication of a corrigendum.

## Figures and Tables

**Figure 1:**
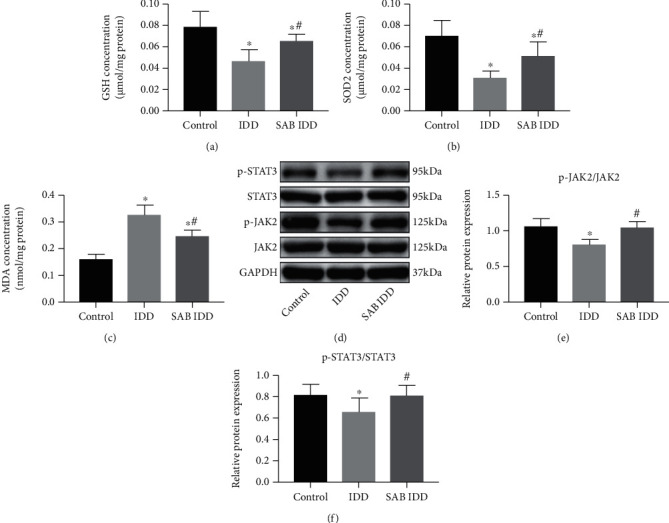
SAB reversed the effects on the antioxidant system induced by puncture injury in vivo that activated the JAK2/STAT3 signaling pathway. Animals were divided into three groups: control group, IDD group, or SAB group. Concentrations of (a) GSH, (b) SOD2, and (c) MDA were measured by assay kits. (d) Expression levels of phosphorylated and total JAK2 and STAT3 in IVDs were measured by Western blotting, and the relative ratios of (e) p-JAK2/JAK2 and (f) p-STAT3/STAT3 were calculated from gray-level values. ^∗^*P* < 0 : 05 compared with the control group; ^#^*P* < 0 : 05 compared with the IDD group. SAB: salvianolic acid B; GSH: glutathione; SOD2: superoxide dismutase 2; MDA: malondialdehyde; GAPDH: glyceraldehyde-3-phosphate dehydrogenase; JAK2: Janus kinase 2; STAT3: signal transducer and activator of transcription 3.

## References

[1] Dai S., Liang T., Shi X., Luo Z., Yang H. (2021). Salvianolic acid B protects intervertebral discs from oxidative stress-induced degeneration via activation of the JAK2/STAT3 signaling pathway. *Oxidative Medicine and Cellular Longevity*.

